# The mouse olfactory peduncle. 2.The anterior limb of the anterior commissure

**DOI:** 10.3389/fnana.2012.00051

**Published:** 2013-01-25

**Authors:** Peter C. Brunjes

**Affiliations:** Department of Psychology, University of VirginiaCharlottesville, VA, USA

**Keywords:** olfactory cortex, olfactory system organization, acetylcholine, serotonin, norepinephrine, orexin, histamine

## Abstract

The central core of the olfactory peduncle [the tissue connecting the olfactory bulb (OB) to the forebrain] includes a white matter tract that extends caudally to the anterior commissure (AC). The purpose of the present study was to examine this “anterior limb of the anterior commissure” (ALAC) to determine if the axons that progress through it segregate on the basis of their point of origin, neurotransmitter type, size, or shape. While local differences in axon density were observed in the ALAC, they were not consistent between samples of the anterior and posterior peduncle, and no other compartmentalization within the tract was observed. The innervation of the caudal olfactory peduncle by neuromodulatory fibers was examined to determine if they enter the region via the ALAC. Cholinergic fibers (CHAT) densely filled the peduncle, followed in order by serotonergic, noradrenergic, histaminergic, and orexinergic processes. Differences in the distribution of the fibers were noted for each system. While each axon type could be observed in the ALAC, it is probable that they enter the peduncle though several routes. Data for axon caliber in the ALAC was compared to information previously collected on the peduncle's other white matter region, the lateral olfactory tract (LOT). Axons in the ALAC were smaller, suggesting that the olfactory system is organized with a fast system for distributing incoming sensory information and a more economical, distributed system for subsequent processing.

The olfactory bulb (OB) is connected to the rest of the forebrain by the region known as the olfactory peduncle (or “retrobulbar area,” e.g., van Alphen, 1969; Figure 1A). All of the structures in the peduncle are involved in processing odor information. In mice, rats and hamsters three large neural regions have components that insert into the peduncle: rostrally the OB extends into the medial peduncle, and caudally the anterior piriform cortex (APC) and olfactory tubercle protrude into the lateral and medial sides, respectively (Figures 1B,C; Davis and Macrides, 1981; Brunjes et al., 2005, 2011). Three areas are primarily contained within the peduncle (Brunjes et al., 2005, 2011). The largest is the anterior olfactory nucleus, (AON; also referred to as the anterior olfactory cortex), which is composed of two substructures: a large ring of cells known as the anterior olfactory nucleus pars principalis (AONpP) and a small, superficial ribbon of neurons known as pars externa. The AON is an important step in olfactory processing. It receives direct input from the OB through axons in the lateral olfactory tract (LOT). It then relays this information both in a feedback link to the ipsilateral OB, where it has the potential to regulate information flow at every synaptic stage of bulb information processing, and in a feedforward pathway to the proximal dendrites of cells in the APC. Importantly, through projections via the anterior limb of the anterior commissure (ALAC), the AON also can regulate the contralateral AON, OB, and APC. The two other structures, the ventral tenia tecta and dorsal peducular cortex, extend into the region dorsal to the peduncle, and have received little attention (Haberly, 2001; Brunjes et al., 2005; Larriva-Sahd, 2012).

**Figure 1:**
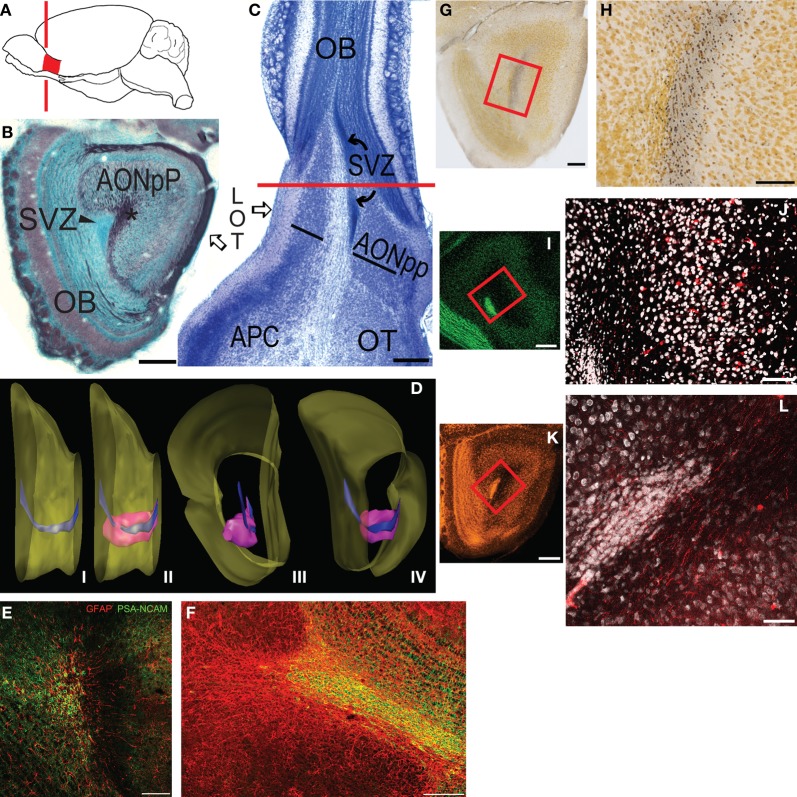
**(A)** Diagram depicting the location of the mouse olfactory peduncle (red). Red line indicates the approximate location of the coronal section in panel **(B)**. **(B)** Coronal Nissl- (blue) and myelin- (black) stained section through the peduncle; medial to left, dorsal at top. **(C)** Horizontal Nissl section through peduncle; lateral to left, rostral at top. Red line is the approximate plane of section in panel **(B)**. Abbreviations: AONpP, pars principalis of the anterior olfactory nucleus; APC, anterior piriform cortex; LOT, lateral olfactory tract; SVZ, subventricular zone (rostral migratory stream); OB, olfactory bulb; OT, olfactory tubercle. Asterisk in **(B)** = anterior limb of the anterior commissure. In **(C)** the AONpP is found on both the lateral and medial side of the ALAC, the white matter region between the two black lines. **(D)** Diagram depicting the SVZ in the peduncle. (i) Medial view of the mouse forebrain; dorsal to top, anterior to right. Surface of the brain is yellow. Most of the OB has been removed from right side. The SVZ (blue) descends from the lateral ventricle at the left of the figure, and extends through the peduncle to the OB. (ii) The SVZ is surrounded by the AONpP (pink). (iii, iv): Posterior and posteromedial perspectives. **(E,F)** The SVZ contains glia (red) upon which neuroblasts (green) travel to the OB as the rostral migratory stream. **(E)** is the central core from a coronal section similar to that seen in **(B)**; **(F)** is from a horizontal section similar to the portion of **(C)** rostral to the red line. **(G–J)**: The ALAC contains axons that cross in the anterior commissure and ascend to the peduncle. **(G,H)** Degeneration staining after AC transection. **(G)** Low power view of peduncle; box outlines region seen in **(H)**. **(H)** Degenerating axons (dark dots) are seen throughout the ALAC. **(I,J)** Results of experiment in which the AC was severed and BDA was applied. **(I)** Low power view of peduncle; box outlines region seen in **(J)**. **(J)** Note scattered filled axons and neuronal somata (red) throughout the ALAC and AONpP. **(K,L)** The ALAC also contains ascending axons from the APC. **(K)** Low power view of peduncle, box outlines region seen in **(L)**. **(L)** Fluororuby-filled fibers in ALAC after injection in APC. Scale Bars: **(B**,**C**,**G**,**I**,**K)**: 250 μm; **(E**,**F)**: 200 μm; **(H**,**J**,**L)**: 100 μm.

The central core of the peduncle contains two structures (Figures 1B,C). The first is the subventricular zone (SVZ), an important landmark for the studies described below. The SVZ is derived from the cells surrounding the olfactory ventricle, which forms at the anterior end of the neural tube during development (Brunjes and Frazier, 1986). In species such as mice and rats, the fluid filled space of the ventricle is reduced with maturation so that the surrounding ependymal cells collapse into a small area. The SVZ contains the “rostral migratory stream,” a pathway by which new neurons born in a proliferative zone in the anterior lateral ventricles move toward the OB (Cummings et al., 1997; Imayoshi et al., 2009; Ming and Song, 2011; Gheusi et al., 2012).

The core of the peduncle also contains a diffuse white matter region containing axons from several sources (Figures 1B,C). Many of the fibers can be traced to anterior commissure (AC), which is found about 2 mm caudal to the peduncle in the mouse. Fibers from neocortical and other regions cross in the posterior aspect of the AC. The anterior side contains projections from the olfactory system: axons originating from cells in the contralateral AON and APC. The bundle travels through the ventral forebrain as the “anterior limb of the anterior commissure” (ALAC). Rostrally directed fibers from the ipsilateral APC also can be found in the ALAC. (Ebner and Myers, 1965; van Alphen, 1969; Broadwell, 1975; Mori and Takagi, 1975; Ryu and Satonishi, 1980; Davis and Macrides, 1981; Jouandet, 1982; Jouandet and Hartenstein, 1983; Illig and Eudy, 2009).

Both the olfactory peduncle and OB receive substantial inputs from caudal brain regions. Indeed, the AON receives projections from at least 27 “non-olfactory” regions (including portions of the hippocampus, cerebral cortex, hypothalamus, amygdala, and thalamus; Brunjes et al., 2005) and it has been suggested that centrifugal inputs into the OB outnumber those coming from the sensory epithelium in the nasal cavity (Macrides and Davis, 1983). It is possible that many of these axons join with the commissural projections as the ALAC proceeds rostrally. The region has been subjected to little formal study. The present paper is an attempt to characterize the ALAC and compare it to data from our previous examination of the LOT, the other major tract in the peduncle.

## Methods

### Animals

C57Bl/6J mice (Jackson Labs; Bar Harbor, Maine) were used. All procedures were performed according to NIH guidelines and protocols approved by the University of Virginia IACUC. Animals were housed in standard polypropylene cages with food (8604, Harlan, Frederick, MD) and water *ad libitum*. The colony was maintained on a 12:12 light:dark cycle in a temperature- and humidity-controlled room.

### Nissl studies

For a general overview of the peduncle (Figure 1), adult male mice were deeply anesthetized with sodium pentobarbital (Euthasol, 0.39 mg drug/gm body weight 150 mg/kg) and perfused transcardially with 0.01 M phosphate buffered saline (pH 7.4; PBS) followed by 4% buffered formaldehyde freshly depolymerized from paraformaldehyde. The brains were removed, post-fixed for several days, embedded in celloidin, cut at 36–60 μm and stained with thionen.

### Myelin staining

A modification of the Schmued (1990) method was used as detailed in Brunjes et al. (2011).

### Anterior commissure transection

Mice were anesthetized and maintained with isoflurane and positioned in a stereotaxic instrument. A midline incision exposed the skull and a 1 mm diameter hole was drilled approximately 1 mm anterior to bregma and 0.5 mm left of the midline. A sterile 27 gauge hypodermic needle was inserted at a 15° angle to the surface of the head, and lowered 4.5 mm such that the end of the needle angled toward the midline. The needle was grasped with forceps and rotated caudally to cut the commissure. The animals were killed 7 days later with an overdose of Euthasol and perfused as above. The tissue was sectioned at 60 μm with a vibratome and processed for degenerating fibers using the “Neurosilver” kit from FD Neurotechnologies (Columbia, MD).

A second group of subjects underwent a similar procedure designed to label axons in the AC with a fluorescent retrograde tracer. The end of a fine nichrome wire was dipped in a concentrated solution of 3000 MW BDA (“microruby”; Invitrogen, Grand Island, NY) and allowed to dry. The wire was then inserted into a 1.0 mm diameter glass pipette until nearly flush with the end. The pipette was introduced into the brain as described above, and the wire extended 1.0 mm. Three days after the surgery the mice were euthanized and perfused as above. The tissue was sectioned on a vibratome and examined with a confocal microscope.

### Anterograde tracer injections

The procedures outlined above were used to label potential projections entering the ALAC from the APC. A pipette containing a 10% solution of 10,000 MW dextran conjugated with rhodamine (“fluororuby”; Invitrogen) in citrate buffer (pH 3.0) was inserted 1.75 mm anterior and 3.2 mm lateral to bregma. The pipette was lowered 4.0 mm and pressure injections (Picospritizer; Parker, Cleveland, OH) of the tracer were made over a 10 min period. After a 5 min diffusion period, the pipette was removed and the wound closed. The animals were killed 2–3 days later with an overdose of Euthasol and perfused as above. Vibratome sections were examined with a confocal microscope.

### Immunostaining and analysis

Fluorescence immunohistochemistry was used to stain free-floating 60 μm-thick vibratome sections. Mice were perfused with paraformaldehyde as above and allowed to postfix for 2 h. [To visualize histaminergic axons, animals were perfused with saline followed by a 4% solution of 1-ethyl-3-(3-dimethylaminopropyl) carbodiimide in PBS, allowed to postfix for 2 h, and then placed into paraformaldehyde overnight; Dacks et al., 2010]. Briefly, the sections were rinsed four times in 0.01 M phosphate buffered saline (PBS pH 7.4). Next, the tissue was incubated in 0.01 M citrate buffer at 80°C (2 × 15 min, Jiao et al., 1999). After cooling at room temp for 5 min, the sections were washed in PBS (2 × 2.5 min), permeablized in 0.03% Triton in PBS (TW: 4 × 5 min), and placed into blocking solution (0.5% normal donkey serum in TW; Jackson ImmunoResearch, West Grove PA) for 1 h. Sections were then placed into primary antibody (Table 1) at least overnight at 4°C (room temp for histamine antibody). They were then washed (PBS 4 × 5 min), and incubated in secondary antibody (1/250–1/450 in TW: Jackson ImmunoResearch. Donkey anti-rabbit: Catalog number, 711-165-152 or 711-545-152; donkey anti-goat: 705-165-147 or 705-545-147; donkey anti-mouse: 715-485-150) for 1 h and washed again (PBS 4 × 5 min). To observe tissue organization some sections were subsequently Nissl-stained (640 nm Neurotrace; Invitrogen: N-21483). Stained tissue was examined on a confocal microscope. In each case, deletion of the primary antibody resulted in no staining.

**Table 1:** **Primary antibodies used**.

**Antigen**	**Immunogen**	**Manufacturer**	**Cat./lot #**	**Species**	**Dilution**
GFAP	Bovine spinal cord isolate	Dako	Z 0334 00076532	Rabbit polyclonal	1/500
PSA-NCAM	Viable meningococcus Group B (strain 355)	Millipore (Temecula, CA)	MAB5324 NG1848932	Mouse monoclonal	1/1000
Orexin A	Peptide mapping at the C terminus of human Orexin A	Santa Cruz (Santa Cruz, CA)	Sc-8070 B1811	Goat polyclonal	1/50
CHAT	Human placental enzyme	Millipore (Temecula, CA)	AB144P NG1780580	Goat polyclonal	1/100
5-HT	Serotonin coulpled to BSA	Immunostar (Hudson, WI)	20080 924005	Rabbit polyclonal	1/1000
NET	22 aa peptide sequence mapped to the first extracellular domain of rat NET	Millipore (Temecula, CA)	AB5066P NG1862054	Rabbit polyclonal	1/1000
Histamine	Synthetic histamine coupled to keyhole limpet hemocyanin w/ carbodiimide linker	Immunostar (Hudson, WI)	22939 1006001	Rabbit polyclonal	1/500

For studies examining neuromodulatory afferents, coronal sections were chosen from a standardized location in the caudal peduncle: the region where AONpP completely encircles the SVZ/ALAC core and Layer 2 is still separate from the overlying cerebral cortex. In order to enhance data diversity at least three animals were studied for each antigen. For each section, montages of the peduncle were produced by tiling 20X images. For each image, two optical sections separated by 3 μm were combined. In order to be able to rank order the degree of staining observed with each antigen, for at least three samples for each antigen, a 350 μm-wide column of tissue from the lateral side of the peduncle extending from the LOT to the ALAC was selected (Figure 3). Image J (Rasband, 1997–2009) was used to transform (to eight bit black and white) and threshold (using the “default” criteria) the images, and then to determine the percentage dark pixels in field (the “area fraction”). In other studies, “horizontal” sections, cut parallel to the ventral surface of the peduncle, were chosen that contained the ALAC as it coursed through the region and into the OB (Figure 4). For the production and analysis of figures, images were acquired and minimally adjusted for brightness and contrast with Adobe Photoshop CS5 and plates were constructed with Adobe Illustrator (San Jose, CA).

### Electron microscopy

Tissue obtained from the same animal used to examine the LOT in the previous study (Brunjes et al., 2011) was employed, and the preparation methods are detailed in that work. Briefly, the mouse was perfused with 2% glutaraldehyde/2% paraformaldehyde and the dissected forebrain post-fixed overnight. Serial vibratome sections (100 μm) were incubated for 30 min in 1% OsO_4_ in 0.1 M phosphate buffered saline and embedded in Epon. Two sections were selected to represent the rostral and caudal olfactory peduncle (Figures 4F and 5F). Ultrathin sections were prepared from these and imaged with a JEOL 1010 TEM at 800X using a 16 megapixel CCD camera. The ALAC at both levels was visualized in composite montages. The boundaries of the ALAC are difficult to define. Data was collected by beginning near the SVZ and proceeding until (1) the region was surrounded by neuronal cell bodies and (2) there was an obvious drop in the density of axonal profiles. Every myelinated profile within the two test regions was outlined on a tablet computer (Lenovo Thinkpad X201). The strategy provided a sample of 36,124 profiles in the anterior section and 50,643 in the posterior area. Image J was used to determine the XY coordinates of all profiles, their minor axis width (a conservative estimate of axon caliber) and to estimate of the circularity of the profiles (using the formula 4π × [area]/[perimeter]^2^; a value of 1.0 indicates a perfect circle, lower numbers represent increasingly elongated shapes) to locate axons oriented obliquely to the plan the section, perhaps indicating that they were changing position within the tract. Matlab (Mathworks, Natick, MA) algorithms were used to produce density plots for each measure.

## Results

### Overview of the core of the olfactory peduncle

The peduncle is a relatively simple region containing well-delineated structures. Figure 1B is an image of a coronal section through the area. In this section, Nissl staining (blue) clearly differentiates the caudal remnant of the OB (left) and the two-layered AONpP. Between the two, in the center of peduncle, is a dense group of small cells, the SVZ, which forms an important landmark used throughout the description of the results below.

The section depicted in Figure 1B is also stained for myelin (black) and shows the two major axon tracts that extend through the peduncle. The LOT, extending down the ventrolateral surface of the forebrain from the peduncle to the entorhinal cortex, is the major output pathway of the OB (Brunjes et al., 2011). The ALAC is the dark region observed in the core of the peduncle lateral to the SVZ. About 2 mm caudal to the peduncle the ALAC exits the AC and descends rostrally in a compact bundle round to oval in cross section. In the caudal peduncle the ALAC is encircled by the cells of the AONpP, APC, and OT. More rostrally the ALAC is encircled entirely by AONpP. Moving still more anteriorly, the dense cell body layers of the AONpP disappears in ventromedial regions, and then progressively recedes both mediodorsally and ventrolaterally until it can only be found under the LOT (see Figure 1, Brunjes et al., 2011). Therefore, myelin staining throughout the peduncle reveals not only the fibers of the ALAC but the deep white matter of the resident neuropils. The older literature has referred this white matter zone as “Layer 3 of the AON” or the “periventricular white matter” (van Alphen, 1969), here the term ALAC will be employed for simplicity.

Figure 1C depicts the region in a section plane that is parallel to the ventral surface of the peduncle, and thus nearly horizontal. The densely packed cells of the SVZ can be seen in the middle of the peduncle (Figure 1C). Lateral to the SVZ, the ALAC is obvious as a large white matter tract. It extends rostrally into the OB and caudally through the length of the section. The AONpP is apparent on either side of the core structures in the peduncle (Figures 1C,D). Caudal to the peduncle the APC and OT occupy the lateral and medial sides of the ALAC, respectively. The SVZ is absent from the caudal portion of the section in Figure 1C as it has gone out of the plane of this section, ascending toward the anterior end of the lateral ventricles. This change in trajectory can be observed in the reconstructions in Figure 1D. The SVZ changes shape as it courses through the peduncle. In the anterior region is a flattened ribbon along oriented dorsoventrally. More caudally it assumes a more compact cross section. Two basic varieties of cells can be found in the SVZ (Doetsch et al., 1997; García-Verdugo et al., 1998). Migrating neuroblasts stain with PSA-NCAM, and the substrate upon which they travel stains for the glial protein GFAP, as seen in coronal (Figure 1E) and horizontal (Figure 1F) sections.

### Composition of the ALAC

Understanding the composition of the deep white matter of the olfactory peduncle is important as it carries axons from higher brain areas that innervate both the peduncle and the more rostrally situated OB.

Several studies were performed to demonstrate that the deep white matter of the peduncle contains fibers that enter from olfactory cortex. Figures 1G–J demonstrate that the ALAC contains axons originating on the contralateral side of the brain. AC transection resulted in degenerating axon profiles that diffusely filled the ALAC (Figures 1G,H). Similarly, applying BDA, a fluorescent retrograde tracer, after severing the commissure labels both scattered fibers and cell bodies in the AONpP (Figures 1I,J). These experiments confirm that the ALAC contains communicating axons from the contralateral olfactory cortices. Depositing fluororuby, an anterograde tracer, in the ipsilateral APC also labeled rostrally directed fibers that extend throughout the region (Figures 1K,L). Axons from these sources do not appear to segregate as they pass through the peduncle, but are diffusely spread through the ALAC.

#### The peduncle is richly innervated by neuromodulatory fibers

**Figure 2:**
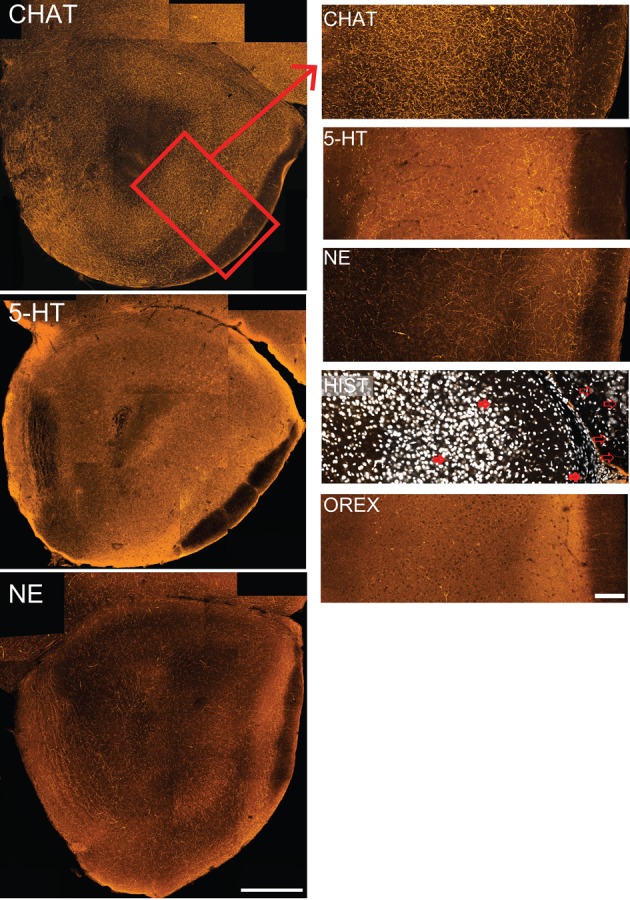
**The olfactory peduncle receives substantial input from neuromodulatory fibers.** Coronal sections indicate different patterns of immunostaining for five neuromodulatory systems. **Left column:** composite confocal images of cholinergic (CHAT), serotonergic (5-HT) and noradrenergic (NE) fibers taken at a standardized location in the caudal peduncle. Histaminergic (Hist) and orexinergic (Orex) staining was too sparse to be seen at low magnifications. Medial to left, dorsal at top. Scale bar: 250 μm. Box in top figure represents the region of the lateral peduncle shown in higher magnification at right. **Right column:** 350 μm wide strips taken from the lateral peduncle for each of the five neuromodulatory systems. Figures include the superficial portion of the ALAC (left) to the LOT and pial surface (right). Few histaminergic fibers are seen in the lateral peduncle (some are indicated by filled arrows), though stained fibers can be observed in the frontal neocortex [unfilled arrows; Nissl staining (white) included for orientation]. Scale bars: 100 μm.

As mentioned above, the olfactory peduncle receives inputs from a large number of “higher” brain regions. Axons from a number of neuromodulatory systems can be found in both the peduncle and OB (e.g., acetylcholine: Záborszky et al., 1986; serotonin: Moore et al., 1978; McLean and Shipley, 1987; norepinephrine: Swanson and Hartman, 1975; Fallon and Moore, 1978; Jones and Yang, 1985; histamine: Auvinen and Panula, 1988; Panula et al., 1989; orexin: Marcus and Elmquist, 2006; Gascuel et al., 2012). However, few studies have compared these pathways within the olfactory peduncle or examined the route by which they enter the region. Coronal sections from a standardized region were chosen for investigation (Figure 2). These sections contained the caudal remnant of the OB (composed primarily of the granule cell layer) on the superficial aspect of the medial peduncle, the AONpP entirely encircling the peduncular core, and, depending on the plane of section, perhaps portions of the ventral tenia tecta, OT, or APC. Comparisons of the tissue revealed obvious differences in the number of stained axonal processes (Figure 2). In order to establish a rough index of density, the “area fraction” of labeled fibers (percentage of pixels in an image field that differ from background) was determined. Cholinergic fibers (CHAT) were about three times as abundant as serotonergic fibers, which, in turn were about twice as plentiful as those stained for norepinephrine (NET). Fibers labeled for histamine and orexin A were relatively rare (≤1% of the pixels).

Along with differing densities, each neuromodulatory system had distinct patterns of axon distribution (Figure 2). While CHAT fibers densely filled the peduncle, regional variations were apparent. CHAT staining was slightly denser in the cell body layer (Layer 2) of AONpP than in the overlying white matter (Layer 1). On the dorsal side, in Layer 1 CHAT immunostained axons were often aligned with the surface of the peduncle. In the granule cell layer of the OB, immunopositive fibers coursed within the spaces between the islands of granule cell bodies, a pattern also seen with tissue stained with NET.

Serotonin and noradrenergic fibers also diffusely filled the peduncle. Both of these neuromodulatory systems had more pronounced immunostaining in Layer 1 of the AONpP than in Layer 2. In the NET stained tissue a band of fibers was often observed in Layer 1a, the region just deep to the LOT. Furthermore, the medial side of the AON often had more NET fibers than the region next to the LOT. OB glomeruli were dense with 5HT fibers and sparse in the in ventral tenia tecta.

Histaminergic and orexinergic fibers were relatively rare in the peduncle. Both were often observed in neocortical and other areas of the sections, affirming that the immunostaining procedures were effective. Most histaminergic fibers were observed in the medial AONpP with few on the lateral side.

#### Do neuromodulatory inputs enter the peduncle via the ALAC?

**Figure 3:**
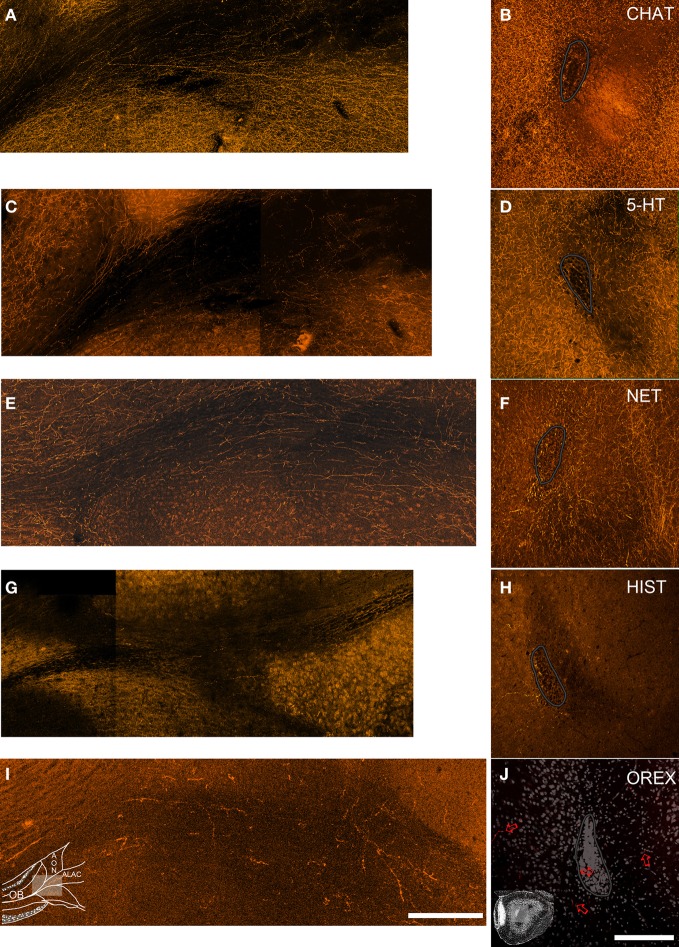
**Neuromodulatory fibers appear to enter the olfactory peduncle both through the ALAC and outside of it. Left column:** horizontal sections (rostral is to the left and medial to the top) through the olfactory peduncle. **Right column:** coronal sections (medial to left, dorsal to top) of the peduncle's core. The SVZ is outlined in gray. Sections were immunostained to visualize cholinergic **(A,B)**, serotonergic **(C,D)**, noradrenergic **(E,F)**, histaminergic **(G,H)**, and orexinergic fibers **(I,J)**. Inset diagrams in panels **(I** and **J)** detail the orientation of the figures. In each of the left panels labeled fibers can be seen extending though the ALAC toward the OB. In the coronal sections (right) a few labeled fibers can be observed in the ALAC, but immunoreactive fibers are also observed within the SVZ and in the medial AONpP (left). Since relatively few orexin positive fibers are seen, this section includes Nissl staining for orientation and arrows indicating a few of the labeled processes. Scale bar = 250 μm.

While it is known that the axons of many of the neuromodulators described above ascend through the medial forebrain bundle (MFB; see Discussion, below), it was of interest to determine if they might ultimately enter the peduncle and perhaps the OB through the ALAC. Several approaches were used. First, if the axons extend through the white matter tract they should be observable in horizontal sections. Indeed, for each of the five antigens, evidence exists that at least some fibers are included in the tract (Figures 3A,C,E,G,I). However, it is difficult to determine if these few sparse processes supply the dense innervation seen in the overlying neuropil. A second approach would be to localize stained processes in coronal sections. However, the small caliber of the fibers, coupled with a plane of section that would cut them perpendicular to their long axes, complicates identification. Indeed, in the coronal sections included in Figure 2 only a small amount of diffuse labeling can be seen in the deep peduncle. As a result, the region of ALAC was revisited in a series of images in which many more optical sections (10–20) separated by only 1 μm were collected and collapsed into a single image (Figures 3B,D,F,H,J). Patterns were similar for all five antigens. While some labeled fibers were apparent in the region of the ALAC, they were small in number and widely separated. For all five systems a few immunopositive axons were also observed within the SVZ. For all antigens except NET relatively dense immunostaining was observed on the medial side of the SVZ, away from the ALAC, which likely represents the anterior extension of the MFB. The density of cholinergic, serotonergic and noradrenergic axons in the tissue surrounding the ALAC obscured determining whether they emerge from a central trunk or simply meander through the tissue.

#### Is there spatial topography within the ALAC?

**Figure 4:**
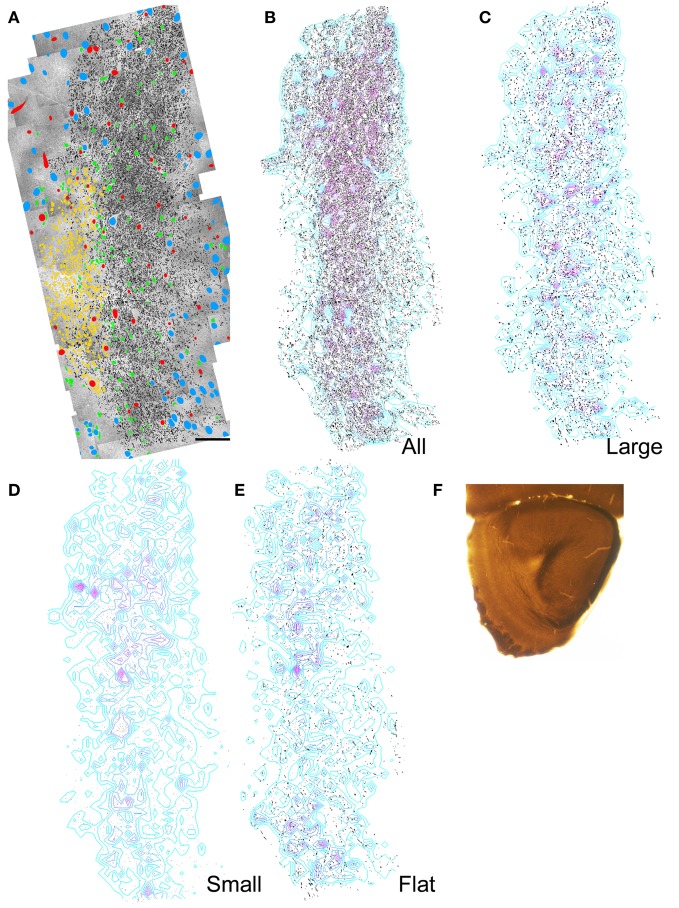
**Results of electron microscopic study of myelinated axons in the anterior olfactory peduncle. (A)** Montages of electron micrographs through the center of the peduncle were constructed. Every myelinated axon in or near the ALAC was outlined and filled with black. The location of neuronal nuclei (blue), blood vessels (red), SVZ cells (yellow), and glial nuclei (green) are marked. Medial to left, dorsal to top. Scale bar: 50 μm. **(B)** Same as panel **(A)** with only the axons shown. Regions of relatively dense axons are depicted by the overlaid contour lines. A dense region of axons parallels the SVZ and encircles it at the dorsal edge (top). **(C)** Distribution of the axons whose minor axes were greater than one SD above the mean. **(D)** Distribution of the axons whose minor axes were less than one SD below the mean. **(E)** Distribution of “flat” axons (see text). **(F)** Low power photomicrograph of the section used in the analysis.

**Figure 5:**
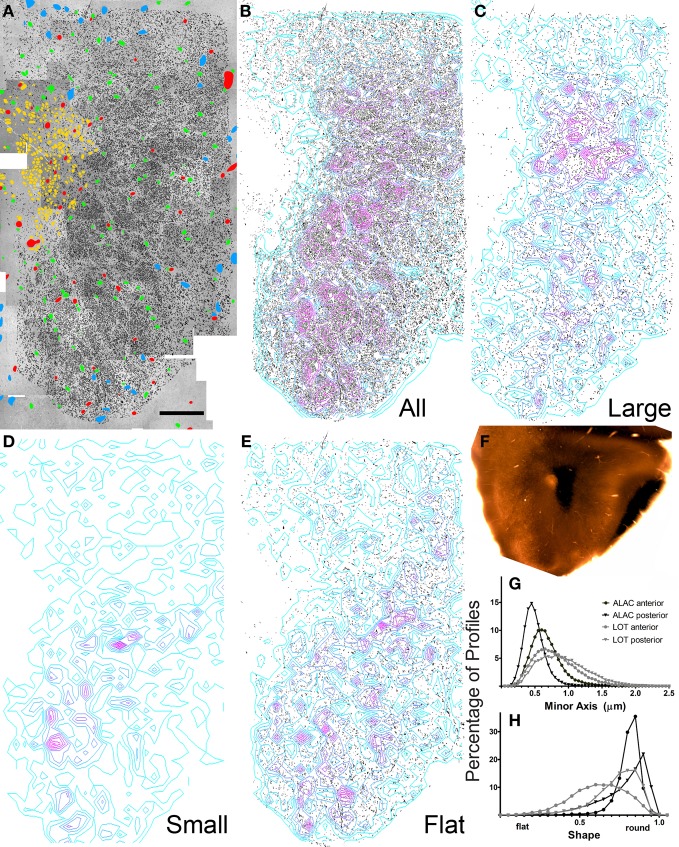
**Results of electron microscopic study of myelinated axons in the posterior olfactory peduncle. (A–F)** See previous figure for details. **(G,H)** Comparison of the percentage of axons exhibiting small to large minor axis sizes and shape factors for both anterior and posterior sections through the ALAC and LOT.

Electron microscopy was used in a manner identical to that reported in a previous examination of the LOT (Brunjes et al., 2011) to determine if there is an internal organization in the ALAC. Two sections representing different rostral-caudal areas were chosen for study (Figures 4F, 5F). In the anterior section (Figures 4A,F) both the white matter and SVZ are roughly parallel flat bands, with the white matter curving slightly around the SVZ on the dorsolateral side. In the posterior region (Figures 5A,F) the SVZ is rounder, and the white matter tract is found lateral and ventral to it. As mentioned above, it is difficult to separate the boundaries of the ALAC from the deep white matter of the AONpP. One solution, defining the white matter region as the zone deep to the neurons that comprise Layer 2 of the AONpP, is confounded by the occasional deep neuronal soma. In the present work, every myelinated axon was traced beginning at the SVZ, proceeding past the first neuronal cell bodies and continuing until there was a drop in the density of axonal profiles.

The composite electron micrographs, marked for neuronal cell bodies, glia, blood vessels, and axons are seen in Figures 4A and 5A. Figures 4B and 5B depict only the axons found in each of the two areas, and thus the sample of over 86,000 profiles that formed the data set. Each of these figures also includes density contours representing areas of concentrated numbers of profiles. Both indicate that there is a dense band of axons just lateral to the SVZ that represents the body of the ALAC. Axons with minor axes greater or less than one standard deviation about the mean were plotted to determine if large or small caliber fibers travel in geographically distinct compartments. In the anterior section, regions dense with large caliber axons were found throughout the tract (Figure 4C), though predominately in the dorsal region. In the posterior area the highest density of large axons was found in a more circumscribed region in the dorsal half of the tract (Figure 5C). Areas with concentrated numbers of small caliber axons were sparse in the anterior section, with only a few seen in the middle of the dorso-ventral extent of the tract (Figure 4D). In comparison, the caudal sample exhibited more regions of dense small fibers dispersed throughout the region (Figure 5D). Finally, flat profiles were concentrated in two zones, the dorsomedial and ventral portions of the white matter in the anterior section (Figure 4E), but in the posterior region were found primarily in the ventral half of the tract (Figure 5E).

Analyses of the total data set indicated several statistical differences between the sample locations (Figures 5G,H). The anterior region exhibited larger mean axon minor axis size than the posterior zone (means = 0.644 vs. 0.465 μm; *t* = 135.42; *P* < 0.0001) and a more varied distribution. A very small, though significant, difference was also observed in axon shape: the average profile in the posterior region was slightly flatter than that observed rostrally (means = 0.783 vs. 0.751 μm; *t* = −36.661; *P* < 0.001).

Axon caliber in the ALAC was significantly smaller than that seen in LOT (anterior sections: 0.644 vs. 0.771 μm; *t* = 48; *P* < 00001; posterior sections: 0.465 vs 0.893 μm; *t* = 204; *p* < 0.0001; Figure 5G). The LOT primarily contains mitral and tufted cell axons, the pojection neurons of the OB. The ALAC it undoubtedly contains a much more diverse axonal population since it carries many centrifugal afferents innervating the peduncle and OB. The flattest axons are seen in the anterior section of the LOT (Figure 5H). This is doubtlessly due to the fact that the tract is near its anterior border and in this region many axons enter the bundle and assume their position.

## Discussion

The work described above is the first to contrast patterns of cholinergic, serotonergic, noradrenergic, histaminergic, and orexinergic innervation in the olfactory peduncle as well as the first to study of the organization of the white matter core of the structure. These results are discussed in turn below.

Studies of a standardized location in the caudal olfactory peduncle revealed that the region is richly innervated by neuromodulatory fibers. Differences in the density of innervation were obvious as cholinergic axons were approximately three times as prevalent as those of the serotonergic system, which were about twice as plentiful as noradrenergic fibers. Orexinergic and histaminergic fibers were relatively scarce. Differences in axonal distribution could be seen along several dimensions. For example, cholinergic fibers were denser in the cellular portion of the AONpP (Layer 2), while serotonergic and noradrenergic fibers were more prevalent in the plexiform layer (Layer 1). The medial side of the peduncle contained more noradrenergic and histaminergic fibers than lateral areas. Distinctive patterns of innervation such as these suggest that various regions in AONpP are differentially influenced by these fibers systems, and thus probably involved in different functions (Brunjes et al., 2005; Meyer et al., 2006).

Unraveling the trajectory the axons take through the peduncle proved difficult. Evidence presented above suggest that axons from each of the systems can be seen coursing through the ALAC, but they are also seen in the SVZ and in the medial AONpP. It is clear that these neuromodulatory axons ascend through the forebrain in the MFB. With axons from more than 50 sources, the MFB is one of the most complex and diffuse tracts in the brain (Saper et al., 1979; Nieuwenhuys et al., 1982). The bundle, subdivided into 13 compartments by Nieuwenhuys et al. (1982) traverses the lateral hypothalamus interspersed with the neuronal elements of the region. Rostrally it becomes dispersed and few studies describe it beyond the OT. Nevertheless, the a, a_1_, and b compartments are found in the medial forebrain in the region of the horizontal limb of the diagonal band just dorsal to the OT, ventral to neostriatum and lateral to nucleus accumbens (Záborszky et al., 1986).

The horizontal limb of the diagonal band is the major source of cholinergic innervation to the OB (Macrides et al., 1981; Záborszky et al., 1986; Niedworok et al., 2012). It seems likely that these axons join in the MFB and then extend into the olfactory peduncle on the medial side of the SVZ (Lohman, 1963). While several papers (e.g., Price and Powell, 1970; Macrides et al., 1981) suggested that a cholinergic projection ascends through or near the LOT, the results reported above indicate that few of these fibers (nor any of the other neuromodulatory systems) could be found in the LOT in the mouse. Macrides et al. (1981) indicated that cholinergic fibers also travel on the medial side of the peduncle in Layer 2 of the AONpP and then “enter the granule cell layer of the MOB directly, or swing dorsally into the internal plexiform layer of the accessory OB in order to reach the middle and anterior part of the MOB (p 504).” The authors termed this route “the final common bulbar pathway” taken by many centrifugal afferents to the OB.

It seems likely that the remainder of the neuromodulatory fibers take a similar route. Serotonergic fibers from the brainstem raphe nuclei have been noted to “… leave the medial forebrain bundle to enter the neostriatum and the external capsule. The continuing group ascends as a loosely arranged series of fascicles in the medial AON, the most superficial of which reach the OB” (Moore et al., 1978, p 421). McLean and Shipley (1987) also suggested that serotonergic fibers enter the peduncle both through a bundle running superficially on the ventromedial side of the peduncle, as well as in the ALAC. Catecholamine containing fibers from three separate systems have been reported to enter the peduncle (Swanson and Hartman, 1975; Fallon and Moore, 1978). Noradrenergic innervation is provided by two regions near the locus coeruleus, one innervating the PC, OT, AON, and OB and another innervating only the OT. Dopaminergic fibers from the lateral ventral tegmental area and medial substantial nigra densely supply the OT with sparse projections to the PC, AON, and OB. Catecholaminergic fibers ascend in the MFB, ultimately joining the rostro-medial continuation of the MFB dorsal to the nucleus of the diagonal band mentioned above. Fallon and Moore (1978) indicated that the AONpP receives a “moderately” dense noradrenergic innervation that is greater on the medial side, that caudal regions are more densely innervated than anterior areas, and that axons in Layer 2 were more plentiful than Layer 1. Only sparse dopaminergic fibers were encountered. Orexinergic axons emerge from cell bodies in the lateral hypothalamus (Marcus and Elmquist, 2006) while histaminergic neurons arise primarily from the caudal tuberal and postmammillary magnocellular hypothalamus (Auvinen and Panula, 1988; Panula et al., 1989), and likely run through the MFB and then into the medial AONpP as well (Nieuwenhuys et al., 1982).

Converging evidence presented above suggests that there is little compartmentalization in the ALAC: axons identified as having the same point of origin (e.g., axons that have crossed in the AC, Figures 1G–J, or originating in the APC, Figures 1K,L), neurotransmitter (Figures 2, 3) or size and shape (Figures 4, 5) do not aggregate consistently within the bundle. The lack of organization on basis of axon size or shape is similar to that recently reported for the mouse LOT (Brunjes et al., 2011). While there have been no previous studies examining the ALAC in other species, there are reports that the LOT of the rat does have internal structure, with larger axons found laterally and smaller ones medially (Price and Sprich, 1975). The apparent lack of structure seen in the ALAC and LOT of the mouse might be (1) due to species differences or (2) the result of the small sample size due to the intensive nature of the data collection.

Differences were encountered when comparing axon caliber between the anterior and posterior regions of the mouse ALAC (means = 0.644 and 0.465 μm). The variation might emerge for several reasons, including differences in the local composition of the tract. For example, in posterior regions the tract might contain more small caliber axons (e.g., neuromodulatory fibers) entering the peduncle from the caudal regions, or perhaps regional differences exist in the number of local axons in the white matter zone. The significance of the observation remains to be determined.

Axon diameter was substantially smaller in the ALAC when compared to the LOT (overall: 0.553 vs. 0.768 μm; Figure 5G). Perge et al. (2012) recently correlated axon size and firing rate in a variety of tissues. They observed that while larger axons can support higher firing rates and thus faster information transfer, the increased size necessitates substantially higher energy expenditures. Since energy stores are finite, axons must deliver information at the lowest acceptable rate. Therefore, large axons should be restricted to situations where information cannot be more efficiently transmitted by spreading it over several small caliber fibers. The LOT carries incoming sensory information that has been sorted and coded by the OB to the olfactory cortex, where presumably meaning is attached (Wilson and Sullivan, 2011). As seen above, the ALAC is involved in feedback relations between contralateral and ipsilateral olfactory cortical centers. The observed differences in axon caliber suggest that the olfactory system is organized with a fast system for initially distributing incoming sensory information and a more economical, distributed system used in subsequent processing.

In summary, the results outlined above are initial observations on the composition of the ALAC, one of two major white matter tracts involved in transmitting olfactory information in the rostral forebrain. The results confirm that the ALAC carries both information from the contralateral and ipsilateral olfactory cortex, and may include at least a few fibers from neuromodulatory systems. Inputs to the olfactory peduncle arise from a number of other locations (Brunjes et al., 2005) whose route of entry remains to be elucidated.

### Conflict of interest statement

The author declares that the research was conducted in the absence of any commercial or financial relationships that could be construed as a potential conflict of interest.
